# Cell adhesion molecule CD44v10 promotes stem-like properties in triple-negative breast cancer cells *via* glucose transporter GLUT1-mediated glycolysis

**DOI:** 10.1016/j.jbc.2022.102588

**Published:** 2022-10-12

**Authors:** Qian Guo, Yaqi Qiu, Yiwen Liu, Yiqing He, Guoliang Zhang, Yan Du, Cuixia Yang, Feng Gao

**Affiliations:** 1 Department of Molecular Biology, Shanghai Jiao Tong University Affiliated Sixth People's Hospital, Shanghai, China; 2 Department of Clinical Laboratory, Shanghai Jiao Tong University Affiliated Sixth People's Hospital, Shanghai, China

**Keywords:** CD44v10, GLUT1, glycolysis, stem cell-like property, triple-negative breast cancer, ALDH, aldehyde dehydrogenase, CSC, cancer stem cell, CD44s, standard CD44 form, CD44v, variant CD44 isoforms, GLUT1, glucose transporter 1, ROS, reactive oxygen species, TNBC, triple-negative breast cancer

## Abstract

Cell adhesion molecule CD44v8-10 is associated with tumor ste0mness and malignancy; however, whether CD44v10 alone confers these properties is unknown. Here, we demonstrated that CD44v10 promotes stemness and chemoresistance of triple-negative breast cancers (TNBCs) individually. Next, we identified that genes differentially expressed in response to ectopic expression of CD44v10 are mostly related to glycolysis. Further, we showed that CD44v10 upregulates glucose transporter 1 to facilitate glycolysis by activating the MAPK/ERK and PI3K/AKT signaling pathways. This glycolytic reprogramming induced by CD44v10 contributes to the stem-like properties of TNBC cells and confers resistance to paclitaxel treatment. Notably, we determined that the knockdown of glucose transporter 1 could attenuate the enhanced effects of CD44v10 on glycolysis, stemness, and paclitaxel resistance. Collectively, our findings provide novel insights into the function of CD44v10 in TNBCs and suggest that targeting CD44v10 may contribute to future clinical therapy.

Triple-negative breast cancer (TNBC) is the most lethal subtype of breast cancers (BrCas), in which the higher frequency of cancer stem cells (CSCs) correlates with a dismal prognosis. Despite the current standard cure modalities, such as surgery, chemotherapy, and radiotherapy, most TNBC patients inevitably suffer from drug resistance and tumor recurrence. Therefore, a better understanding of the molecular basis for TNBC progression is urgently needed to discover new and more effective therapeutic targets for patients.

CD44 is a major adhesion molecule and has been implicated in various biological behaviors, such as cell differentiation and cell motility, as well as tumor growth and metastasis (1, 2). The human CD44 gene consists of 19 exons and undergoes extensive alternative splicing that generates two families of isoforms, the standard CD44 form (CD44s) and the variant CD44 isoforms (CD44v). In contrast to the ubiquitous expression of CD44s, CD44v, which contains one or more variant exons, seems to be restricted to subpopulations endowed with stem cell potential and tumor development. Among CD44v, CD44v10-containing isoforms encompass a group of isoforms, such as CD44v8-10, CD44v3-10, and CD44v2-10, which have been identified as the most abundant variants (2, 3). It has also been reported that CD44v10-containing isoforms are involved in regulating metastasis, stemness, and chemoresistance of several cancers (4, 5, 6). Blockade of CD44v10 could downregulate the expressions of all isoforms containing exon-v10 and consequently delay tumor growth and metastasis (7, 8, 9). Therefore, targeting CD44v10-positive cancer cells arises as a promising cancer therapy option. However, the question of whether it is CD44v10 alone or CD44v10 cooperates with its related isoforms together that influences cancer behaviors remains unanswered.

In this study, we first demonstrated that CD44v10 is preferentially expressed in TNBC patients and correlates with tumor progression. Next, we generated a breast cancer cell line transfected with CD44v10 cDNA and identified that glycolysis-related genes were significantly upregulated in response to CD44v10 overexpression. As reported before, abnormal glycolysis metabolism is usually adopted by CSCs, such as glucose uptake, glycolytic enzyme expression, and lactate production which are elevated in CSCs as compared with their differentiated offspring (10, 11, 12, 13). Moreover, certain CD44v isoforms, such as CD44v3, CD44v6, and CD44v8-10, could act as CSCs markers and play critical roles in promoting the properties of CSCs (5, 14, 15). These findings raise the possibility that CD44v10 may be involved in regulating the stem-like features. We next performed transcriptomic analysis and determined that CD44v10 enhances glycolysis through glucose transporter 1 (GLUT1) upregulation, which could maintain TNBC cells stem-like properties and confer paclitaxel (PTX) resistance. Notably, silencing of GLUT1 could markedly suppress the enhanced effects of CD44v10 on glycolysis and stem-like properties. Taken together, our findings suggested that CD44v10 could promote BrCas stemness individually without cooperating with other CD44 variants. The underneath mechanism of CD44v10-GLUT1 may point to CD44v10 as a specific therapeutic target to the clinic in TNBC.

## Results

### CD44v10 is preferentially overexpressed in TNBC patients

**Figure 1:**
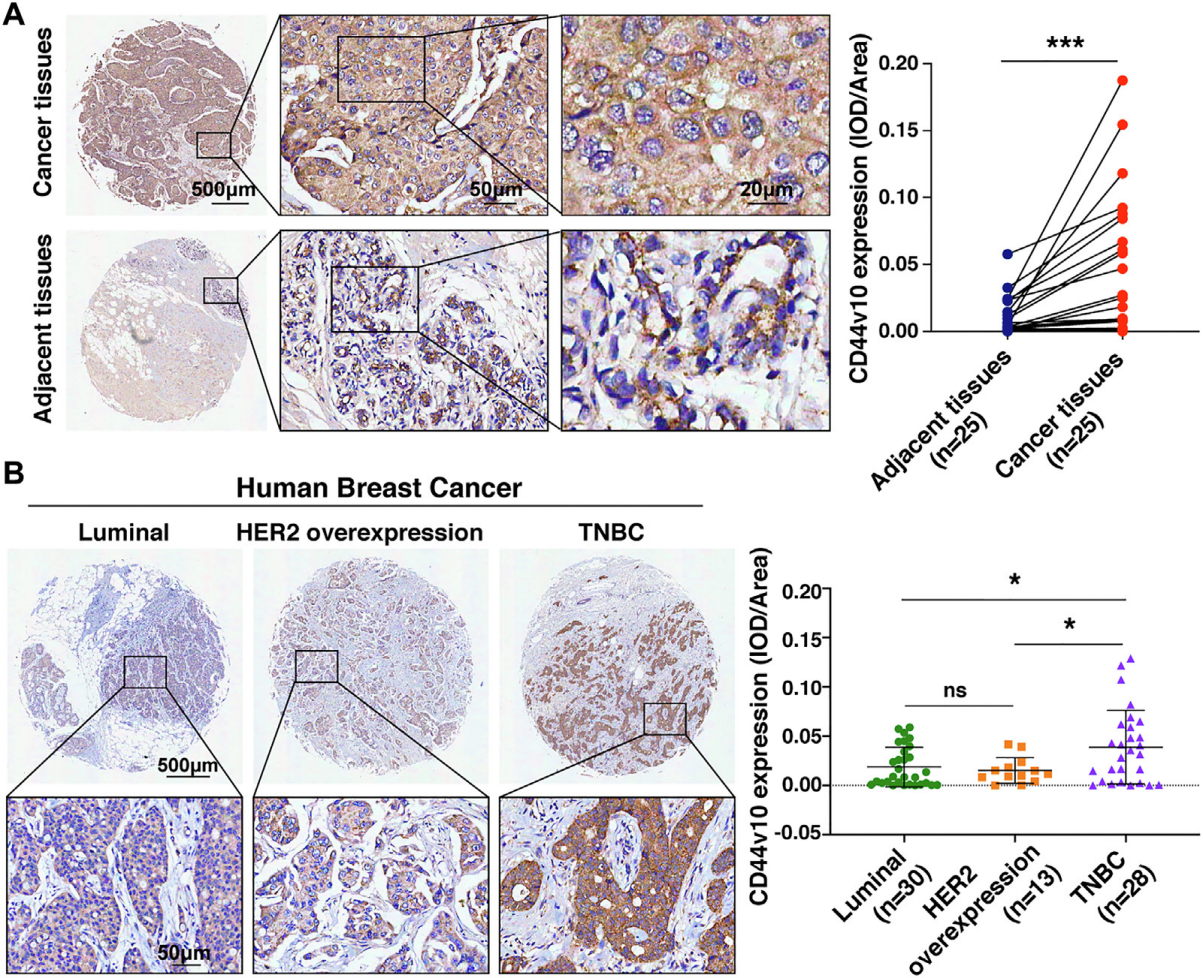
**CD44v10 is preferentially overexpressed in TNBC tissues**. *A*, CD44v10 expression in BrCa tissues and adjacent normal tissues is shown by immunohistochemistry (IHC). Representative IHC images (*left*) and analysis of expressions (*right*) for CD44v10 in 25 pairs of BrCa and matched peritumoral tissues. Statistically significant differences were determined using *t*-tests. *B*, representative IHC images (*left*) and analysis of expression (*right*) for CD44v10 in a tissue microarray, including luminal BrCas (n = 30), BrCa patients with HER2 overexpression (n = 13), and TNBCs (n = 28). The mean of integrated optical density (IOD) was calculated by the IOD divided by the valid area. Bars represent the mean ± SD values. The ns indicates no significance, ∗*p* <0.05, ∗∗∗*p* <0.001. TNBCs, triple-negative breast cancers.

**Figure 2:**
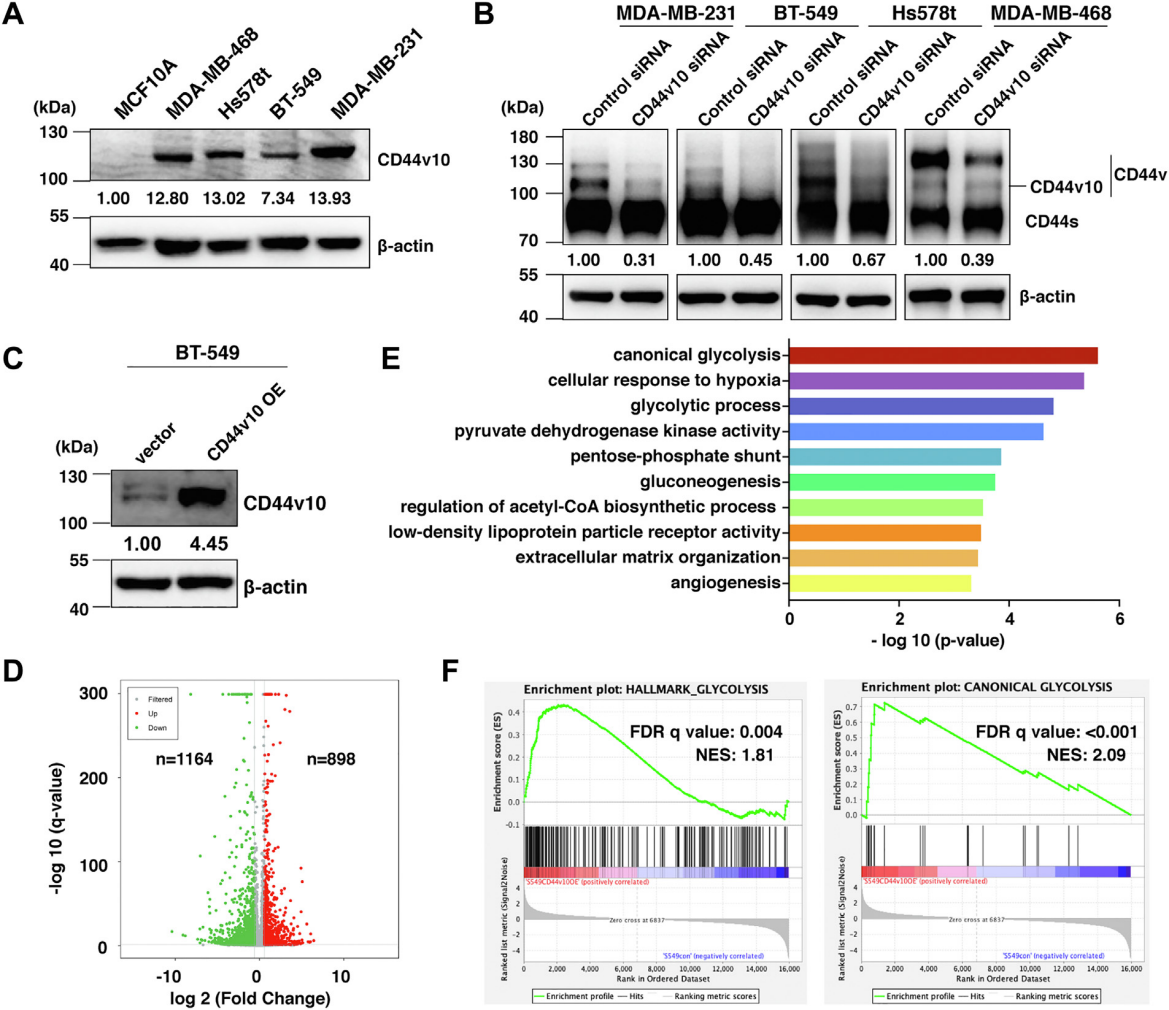
**Analysis of transcriptional changes upon CD44v10 overexpression**. *A*, CD44v10 expression levels of human normal breast epithelial cells (MCF10A) and TNBC cells (MDA-MB-468, Hs578t, BT-549 and MDA-MB-231) were analyzed by Western blotting. β-actin was used as the loading control. *B*, CD44 expression levels of TNBC cells after CD44v10 siRNA transfection were analyzed by Western blotting. *C*, overexpression (OE) efficiency of CD44v10 was evaluated by Western blotting. *D*, volcano plot of log_2_ fold changes *versus* -log_10_ q value shows transcriptional differences between BT-549 vector and BT-549 CD44v10 overexpression cells. Vertical lines represent the 1.5-fold change cut off and the horizontal lines indicate the 0.05 q value cut-off. Upregulated and downregulated genes are highlighted in *red* and *green*, respectively. *E*, gene ontology (GO) analysis of upregulated differentially expressed genes in terms of biological processes. *F*, gene set enrichment analysis (GSEA) enrichment plots of the hallmark of glycolysis and canonical glycolysis gene sets in BT-549 CD44v10 overexpression cells compared with BT-549 vector group. FDR, false discovery rate, NES, normalized enrichment score; TNBCs, triple-negative breast cancers.

To investigate the potential role of CD44v10 in BrCas, we first analyzed its expression in a cohort of 25 paired BrCas tissues. The results of immunohistochemical staining showed that CD44v10 levels were dramatically elevated in cancer tissues than adjacent noncancerous tissues (Fig. 1*A*). Next, to identify the correlation between CD44v10 and BrCas heterogeneity, we detected CD44v10 expressions among distinct molecular subtypes by using another cohort, which contains 30 cases of luminal BrCas, 13 cases of BrCas with HER2 overexpression, and 28 cases of TNBCs. Our results revealed that CD44v10 expression was significantly elevated in TNBCs compared with that in HER2-overexpression and luminal BrCas (Fig. 1*B*), implicating an important role of CD44v10 in the carcinogenesis of TNBCs. Similar results were obtained by utilizing several human BrCa cell lines showing that CD44v10 expression was higher in four TNBC cell lines (MDA-MB-468, MDA-MB-231, Hs578t, and BT-549) than human normal breast epithelial cells (MCF 10A) (Fig. 2*A*). Subsequently, we analyzed the correlation between CD44v10 and clinicopathological features in cohort 1 and cohort 2 BrCa patients. We did not find an obvious correlation of CD44v10 expression with age, tumor size, and lymph node status; however, tumors with high CD44v10 expression had higher histopathological grades and more advanced Tumor, Lymph Node, Metastasis stages (Table 1). Moreover, Kaplan–Meier curves were measured in an additional tissue microarray containing 135 human BrCas to identify the correlation between CD44v10 and survival time. The results revealed that BrCas patients with higher expression of CD44v10 showed shorter overall survival time (Fig. S1). Collectively, these data suggested that CD44v10 is preferentially expressed in TNBC patients and may play a vital role in the development of human BrCas.

**Table 1:** Correlation of CD44v10 expression with the clinicopathological feature of primary breast cancer patients

Characteristic parameter	N	CD44v10 expression (mean IOD) ∗10^-2^	Median	*p-value*
Age				0.604
< 50 years	24	2.622 ± 3.987	0.685	
≥ 50 years	72	3.036 ± 3.007	2.078	
Histopathological grade				**0.035**
I-II	63	2.417 ± 2.845	1.304	
III	33	3.894 ± 3.757	2.965	
Tumor size				0.843
≤ 2 cm	15	3.08 ± 2.973	2.273	
>2 cm	81	2.896 ± 3.315	1.370	
Lymph node status				0.950
Negative	31	2.894 ± 2.306	2.964	
Positive	65	2.939 ± 3.633	1.302	
TNM stage				**0.006**
Early stage (I-II)	64	2.283 ± 2.690	1.259	
Advanced stage (III)	32	4.028 ± 3.877	3.187	

The bold value indicates a significant difference.

Abbreviations: N, number; TNM, Tumor, Lymph Node, Metastasis.

### Analysis of transcriptional changes upon CD44v10 overexpression

Our previous study showed that expressions of all isoforms containing exon-v10 were significantly decreased in MDA-MB-231 cells after CD44v10 siRNA transfection (7). In the present study, similar results were obtained by using other TNBC cell lines (BT-549, Hs578t, and MDA-MB-468) (Fig. 2*B*). To determine the roles of CD44v10 itself in TNBC progression, we established a stable cell line by transfection of CD44v10 cDNA into BT-549 cells which contains little endogenous CD44v10 (Fig. 2*C*). The global transcriptome profiles in BT-549 cells transduced with vector or CD44v10 were examined by RNA sequencing. Differential expression analysis (fold change >1.5, *p* < 0.05) highlighted 898 and 1164 genes significantly upregulated and downregulated in BT-549 CD44v10 cells compared with vector samples, respectively (Fig. 2*D*). Functional annotation revealed that genes upregulated in CD44v10 overexpression cells were significantly enriched for pathways related to glycolysis, gluconeogenesis, and angiogenesis (Fig. 2*E*). To further extend this observation, we applied gene set enrichment analysis and confirmed a significant positive enrichment of glycolysis gene sets in BT-549 CD44v10 group compared with BT-549 vector cells (Fig. 2*F*). Taken together, our results suggested that CD44v10 may be involved in glycolysis.

### Regulation of CD44v10 impacts glycolytic processes of TNBC cells

**Figure 3:**
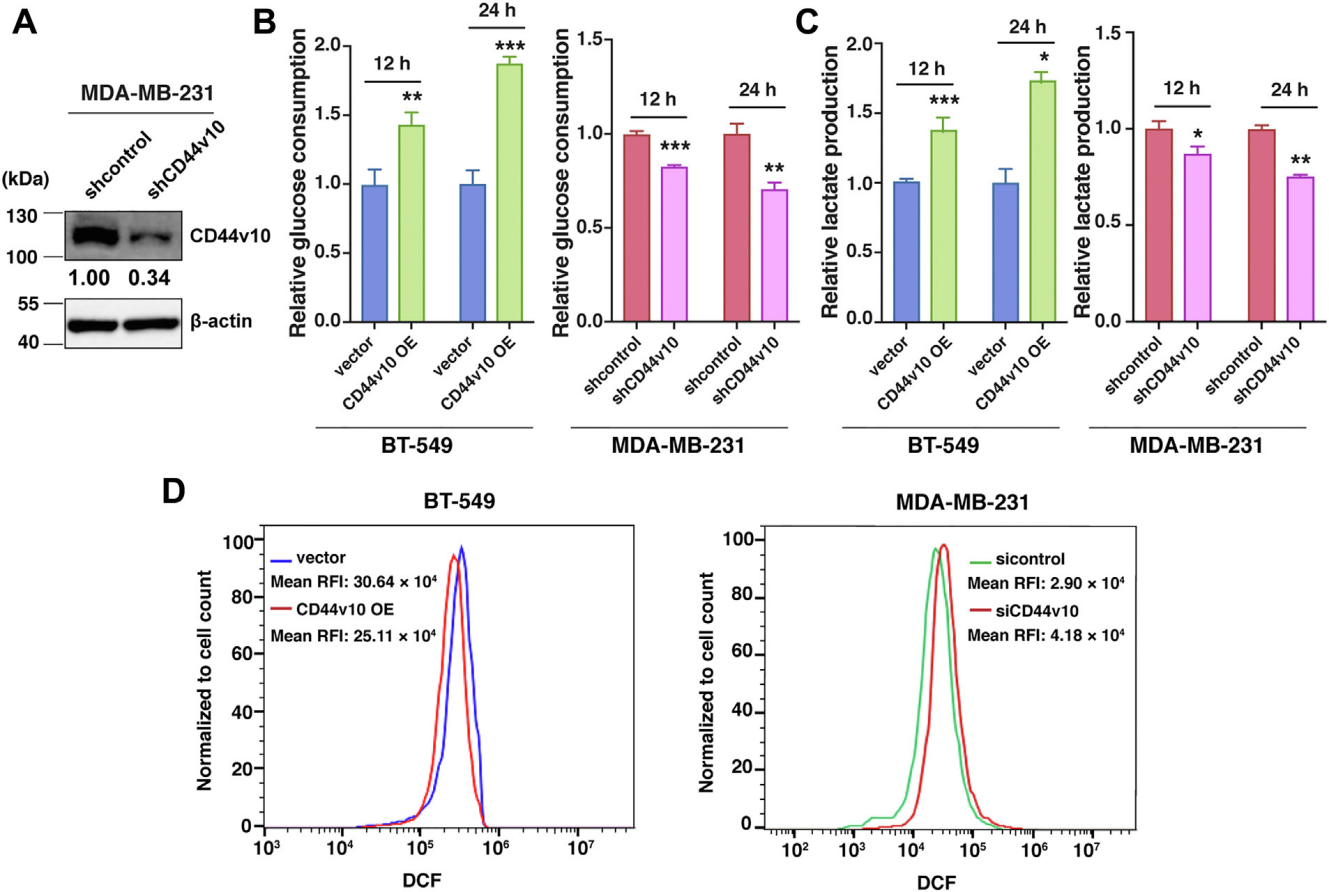
**Regulation of CD44v10 impacts glycolytic processes of TNBC cells.***A*, knockdown efficiency of CD44v10 in MDA-MB-231 cells was evaluated by Western blotting. *B*, analysis of glucose consumption in cells cultured for 12 and 24 h. The means ± SD of relative fold changes from triplicate experiments were plotted. *C*, analysis of lactate production in cells cultured for 12 and 24 h. *D*, cells were stained with 2',7'- dichlorodihydrofluorescein-diacetate (DCFH-DA), and then subjected to flow cytometry analysis to measure the cellular ROS level. The mean relative fluorescence intensity (RFI) values are shown. ∗*p* <0.05, ∗∗*p* <0.01, ∗∗∗*p* <0.001. ROS, reactive oxygen species; TNBCs, triple-negative breast cancers.

To validate the RNA sequencing results, we next examined the changes in glycolytic metabolism altered by CD44v10 expression. In addition to BT-549 cells, we also knocked down CD44v10 in MDA-MB-231 cells, which contain a high level of endogenous CD44v10 (Fig. 3*A*). Given that glycolytic phenotype is characterized by increased glucose consumption and lactate secretion along with reduced reactive oxygen species (ROS) levels (16), we examined whether alteration of CD44v10 expression affects levels of glucose, lactate, and ROS. Our results indicated that ectopic expression of CD44v10 increases glucose consumption and lactate secretion as well as decreases intracellular ROS level, while the knockdown of CD44v10 reverses such effects (Fig. 3, *B*–*D*). These findings suggested that CD44v10 could promote the glycolytic processes of TNBC cells.

### CD44v10 facilitates glycolysis by upregulating GLUT1 expression

**Figure 4:**
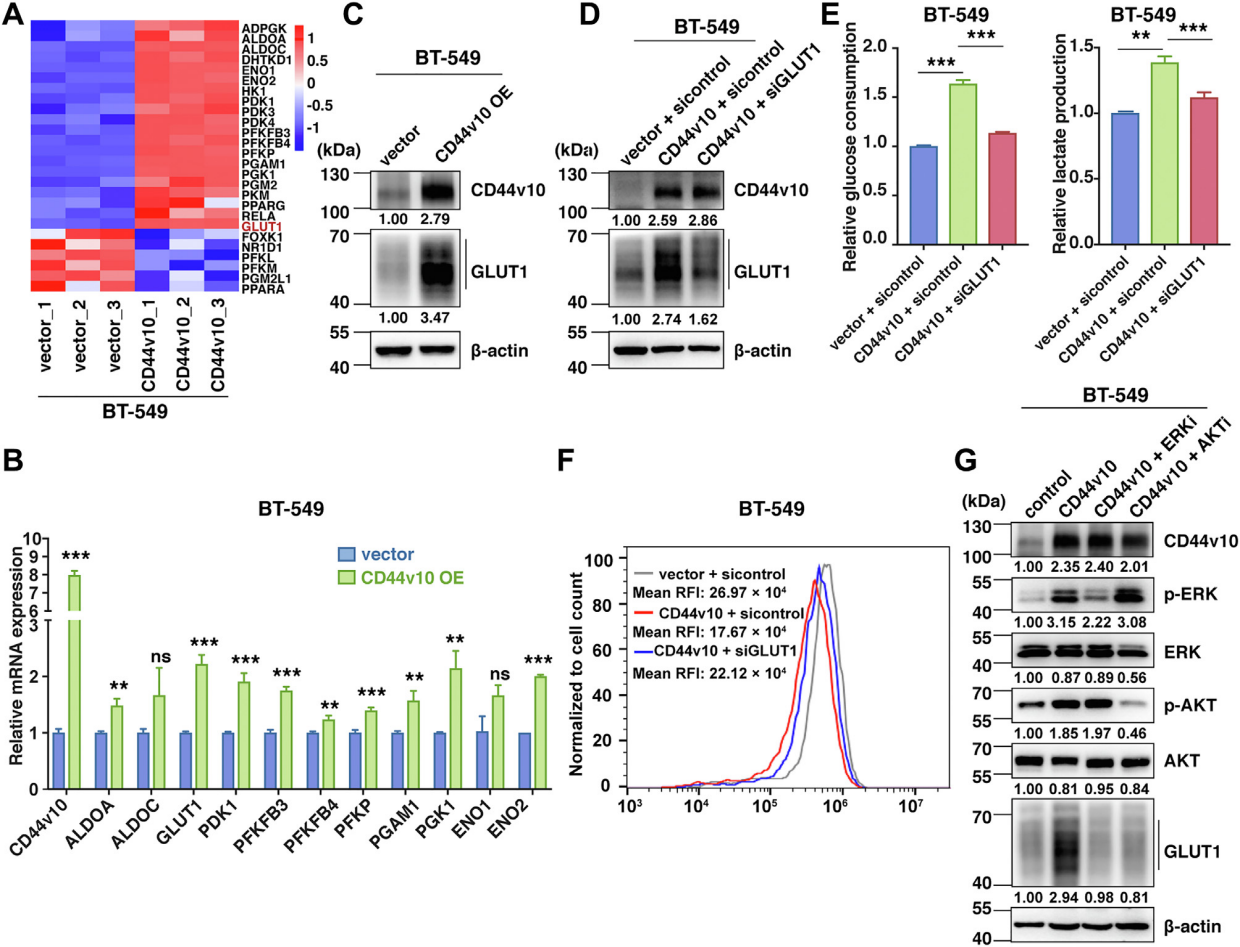
**CD44v10 facilitates glycolysis by upregulating GLUT1 expression**. *A*, heatmap shows expression profiles of the glycolysis-related genes in BT-549 CD44v10 overexpression cells compared to BT-549 vector cells. *Red colors* indicate upregulation, and *blue* indicates downregulation. *B*, expression validation of candidate genes from heatmap by qPCR. The means ± SD of relative fold changes from triplicate experiments were plotted. β-actin was used as the control. The *p* values were calculated by paired Student’s *t* test. *C*, analysis of GLUT1 expression by Western blot in CD44v10 OE cells. *D*, knockdown efficiency of GLUT1 was evaluated by Western blotting in CD44v10 OE cells. *E*, the effects of GLUT1 on glucose consumption and lactate production in BT-549 cells. *F*, the effects of GLUT1 on ROS levels in BT-549 cells. *G*, relative levels of phosphorylation of ERK, AKT, and GLUT1 proteins in BT-549 CD44v10 overexpression cells pretreated with ERK (U0126, 20 μM) or AKT inhibitor (LY294002, 20 μM). The ns indicates no significance, ∗∗*p* <0.01, ∗∗∗*p* <0.001. GLUT1, glucose transporter 1; ROS, reactive oxygen species.

To gain insight into the mechanism by which CD44v10 potentiated TNBC glycolytic phenotypes, the gene expression patterns of key glycolytic enzymes in the glucose metabolic pathway were compared between BT-549 vector and BT-549 CD44v10 overexpression groups. As expected, most of the glycolysis-related genes were upregulated in CD44v10 overexpression cells (Fig. 4*A*). We further validated these gene expression levels by qPCR assay and selected three candidates (GLUT1, PGK1, and ENO2) that were robustly upregulated upon CD44v10 overexpression (Figs. 4*B* and S2). Next, the protein expressions of the three genes were detected, and the results showed that GLUT1, the key rate-limiting factor of cellular glucose uptake, was significantly increased in CD44v10 overexpression cells, while no obvious changes of PGK1 and ENO2 were observed upon CD44v10 ectopic expression (Figs. 4*C* and S2). These results suggested that GLUT1 may be principally responsible for the CD44v10-induced glycolysis.

To substantiate the contribution of GLUT1 in CD44v10-mediated glycolysis, we next investigated whether the knockdown of GLUT1 affects the activated glycolytic processes caused by CD44v10 overexpression. The CD44v10 overexpression cells were transfected with control siRNA or GLUT1 siRNA (Fig. 4*D*). As shown in Fig. 4*E*, the silence of GLUT1 attenuated the increased levels of glucose consumption and lactate production induced by CD44v10 overexpression. A similar result was also obtained on ROS generation, which showed that the knockdown of GLUT1 rescued the reduced ROS level caused by CD44v10 overexpression (Fig. 4*F*). Further, we explored the mechanism by which CD44v10 induces GLUT1 expression. As we previously reported that CD44v10 could activate MAPK/ERK and PI3K/AKT signaling pathways (7) and others have indicated that such signaling could induce the GLUT1 expression (17, 18), we next asked whether MAPK/ERK and PI3K/AKT cascades are engaged in the CD44v10-induced upregulation of GLUT1. As expected, the inhibition of the signaling pathways could impair the CD44v10-triggered GLUT1 upregulation (Fig. 4*G*). Collectively, the data suggested that CD44v10 may upregulate GLUT1 *via* activating MAPK/ERK and PI3K/AKT signaling pathways in facilitating glycolysis.

### CD44v10 promotes CSCs properties of TNBC cells

**Figure 5:**
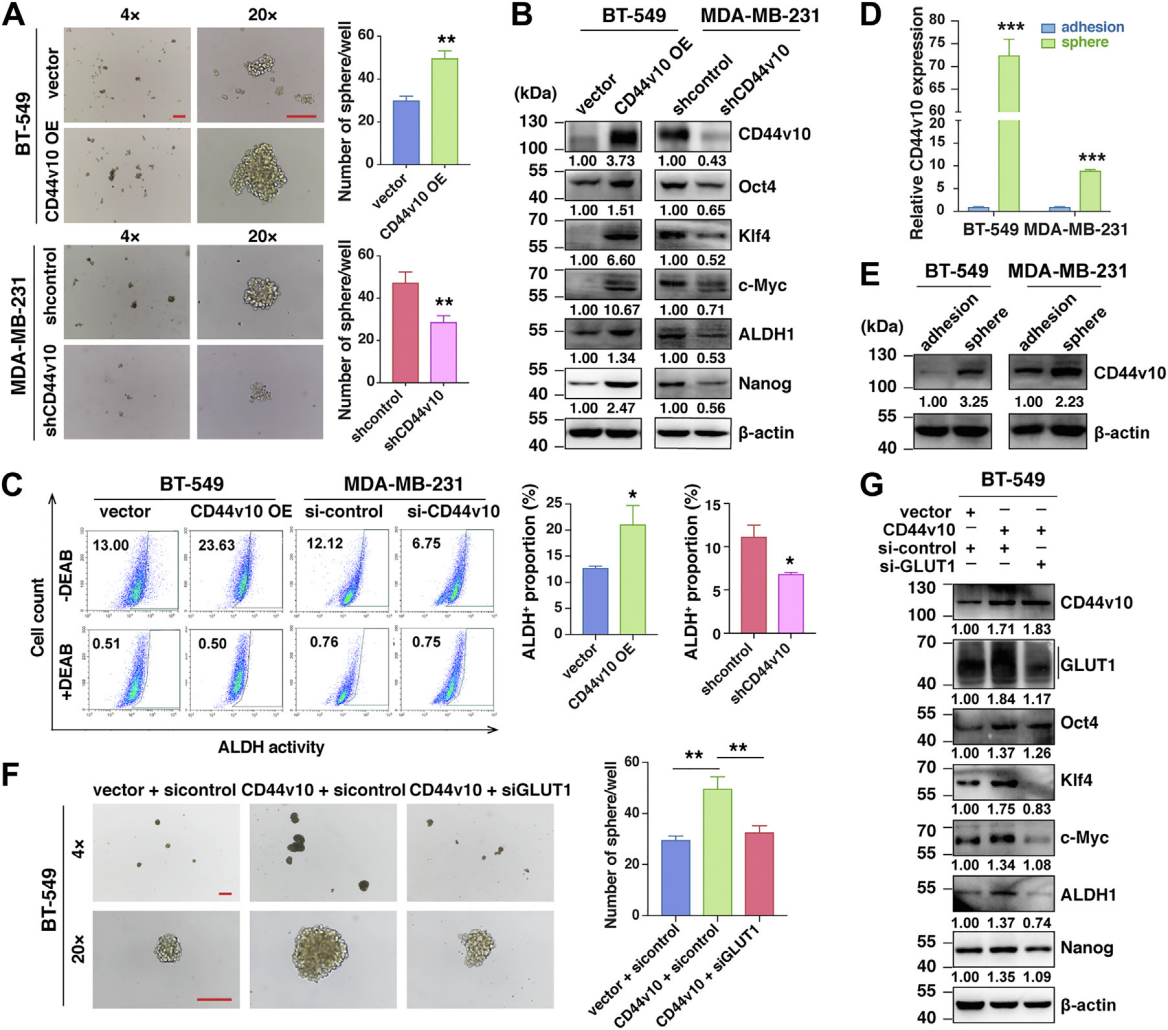
**CD44v10 promotes CSC properties of TNBC cells**. *A*, the effects of CD44v10 overexpression or inhibition on sphere-forming ability were detected by sphere-forming assays. Scar bars, 400 μm for (4×) and 200 μm for (20×). *B*, the effects of CD44v10 overexpression or knockdown on protein expressions of a panel of stemness-related genes (Oct4, Klf4, c-Myc, ALDH1, and Nanog) were analyzed by Western blotting. *C*, ALDH-positive population was detected by flow cytometry (*left*). The proportions were calculated from triplicate independent experiments (*right*). *D* and *E*, mRNA (*D*) and protein (*E*) levels of CD44v10 were tested in spheroid cells and adherent cells by qPCR (*D*) and Western blotting (*E*), respectively. *F*, the effects of GLUT1 on sphere-forming ability of BT-549 cells. Scar bars, 400 μm for (4×) and 200 μm for (20×). *G*, the effects of GLUT1 on the expressions of CSC-related genes in BT-549 cells by Western blotting. ∗*p* <0.05, ∗∗*p* <0.01, ∗∗∗*p* <0.001. ALDH, aldehyde dehydrogenase; CSC, cancer stem cell; GLUT1, glucose transporter 1; TNBC, triple-negative breast cancer.

Metabolic reprogramming in cancer cells has been reported to support the maintenance of CSCs properties (12). Additionally, it is now accepted that CD44v has been identified as one of the important markers of CSCs in many malignant tumors, which prompted us to further investigate whether CD44v10-activated glycolysis could contribute to the stemness properties of TNBC cells. After carrying out sphere formation assays, we found that CD44v10 overexpression remarkably increased the formation of spheroids compared to the control cells in BT-549 cells, whereas CD44v10 knockdown greatly hindered the sphere numbers (Fig. 5*A*). As the knockdown of CD44v10 could interfere the expressions of all CD44v10-containing isoforms, we next performed a rescue experiment by re-expressing CD44v10 in MDA-MB-231 shCD44v10 cells to further verify the role of individual CD44v10 in mediating TNBC stemness. The results showed that the re-expression of CD44v10 could restore the reduced sphere-forming ability of MDA-MB-231 caused by CD44v10 knockdown (Fig. S3). In addition, Oct4, Klf4, c-Myc, aldehyde dehydrogenase (ALDH)1, and Nanog are essential renewing and pluripotent regulators of stem cells and CSCs (19). We next examined the effects of CD44v10 on the expressions of these CSC-related factors and found that their expressions were upregulated in BT-549 CD44v10 overexpression cells while downregulated in the MDA-MB-231 shCD44v10 group (Fig. 5*B*). Furthermore, ALDH-positive populations are believed to contain breast CSC subsets (20). In line with these observations, the proportions of ALDH-positive cells increased from 13.00% to 23.63% in BT-549 CD44v10 overexpression compared with control cells, whereas it decreased from 12.12% to 6.75% in MDA-MB-231 siCD44v10 compared with sicontrol cells (Fig. 5*C*). To further corroborate the function of CD44v10 on stemness, we then analyzed the expression of CD44v10 in TNBC cell lines grown as either adherent monolayers or nonadherent spheres enriched in CSCs. The results showed that both mRNA and protein levels of CD44v10 were elevated in spherical cells compared to their adherent cells (Fig. 5, *D* and *E*).

To confirm the contribution of GLUT1 in CD44v10-mediated stemness, we investigated whether the knockdown of GLUT1 could attenuate the enhanced stemness caused by CD44v10 overexpression. As shown in Fig. 5, *F* and *G*, GLUT1 knockdown abrogated the upregulation of tumorsphere and stemness gene expression induced by elevated expression of CD44v10 in BT-549 cells. Taken together, these results indicated that CD44v10 confers TNBC stem cell-like phenotype by upregulating GLUT1 expression.

### CD44v10 correlates with PTX sensitivity of TNBC cells

**Figure 6:**
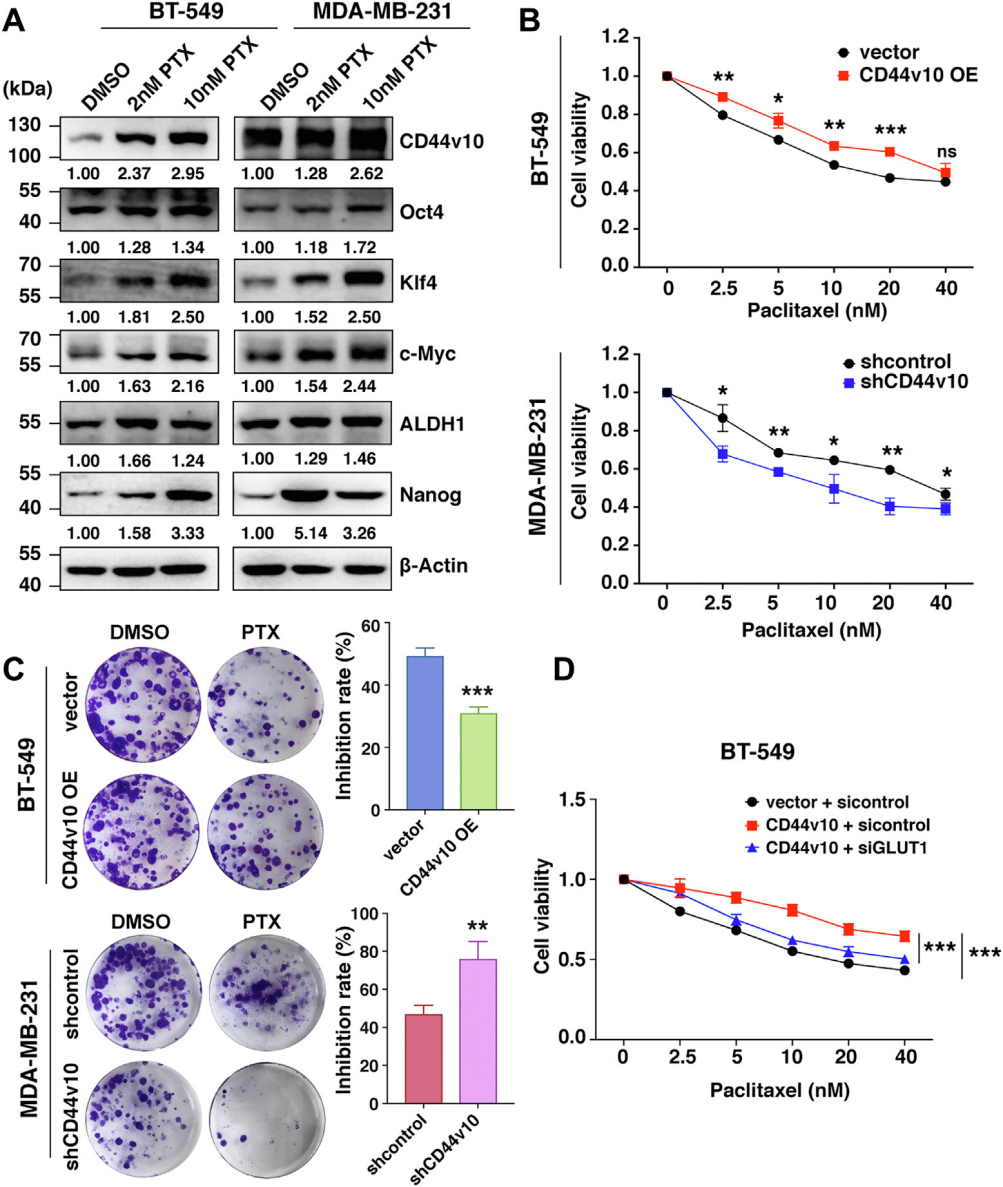
**CD44v10 correlates with paclitaxel sensitivity of TNBC cells**. *A*, the effects of paclitaxel (PTX) treatment on the protein expressions of CD44v10 and stemness-related genes in BT-549 and MDA-MB-231 cells by Western blotting. *B*, CCK-8 assay is used to evaluate the chemosensitivity of BT-549 and MDA-MB-231 cells to different concentrations of PTX after 48 h treatment. *C*, the effects of CD44v10 on the sensitivity of BT-549 and MDA-MB-231 cells to PTX by colony formation assay. *D*, the effects of GLUT1 on the sensitivity of BT-549 cells to PTX by CCK-8 assay. Statistical significance was determined by one-way ANOVA. Data are shown as the mean ± SD. The ns indicates no significance, ∗*p* <0.05, ∗∗*p* <0.01, ∗∗∗*p* <0.001. GLUT1, glucose transporter 1; TNBC, triple-negative breast cancer.

CSCs are inherently responsible for tumor resistance to conventional chemotherapy. PTX is a predominantly used systemic treatment for TNBC patients. In light of the crucial role of CD44v10 in CSCs regulation, we sought to investigate whether the CD44v10 isoform is associated with chemosensitivity to PTX. Western blot results showed that PTX treatment increased the expression of CD44v10 along with CSC-related genes (Oct4, Klf4, ALDH, c-Myc, and Nanog) in a dose-dependent manner in both BT-549 and MDA-MB-231 cell lines (Fig. 6*A*). In addition, concerning chemical sensitivity, the cytotoxic effects of different concentrations of PTX on CD44v10-regulated cells and their respective control cells were detected after 48 h treatment of PTX by CCK-8 assays. The results indicated that the upregulation of CD44v10 in BT-549 cells resulted in resistance to PTX and its knockdown in MDA-MB-231 cells enhanced drug sensitivity to PTX-induced growth inhibition (Fig. 6*B*). To further verify the role of individual CD44v10 in regulating PTX sensitivity, we performed rescue experiments and found that the re-expression of CD44v10 could attenuate the increased PTX sensitivity caused by CD44v10 knockdown (Fig. S4). Similar results were also obtained in plate colony formation assay (Fig. 6*C*). These findings indicated that CD44v10 is associated with tumor resistance to chemotherapy. To confirm the contribution of glycolysis in CD44v10-mediated PTX sensitivity, we downregulated GLUT1 in BT-549 CD44v10 overexpression cells and found that the knockdown of GLUT1 could rescue the reduced PTX sensitivity caused by CD44v10 overexpression (Fig. 6*D*).

## Discussion

CD44v10-containing isoforms have been concerned for their overt activities in tumor growth, metastasis, and stemness. However, little is known about whether CD44v10 itself plays role in cancer progression compared to others such as CD44v6 and CD44v3. In this study, we demonstrated that CD44v10 independently promotes stem-like properties and decreases PTX sensitivity of TNBC cells at *in vitro* experiments. More importantly, we reported a novel mechanism of CD44v10 on BrCas stemness through glucose metabolism, which is mediated by GLUT1-induced glycolysis.

Although high-level expression of CD44v10 has been observed in several cancers and correlates with a worse prognosis (21, 22, 23), there has limited information regarding the carcinogenic role of CD44v10 in BrCas. Herein, we evaluated the clinical significance of CD44v10 in breast cancer patients and found that CD44v10 was preferentially expressed in TNBC patients compared with HER2-positive or luminal BrCas. Notably, BrCa patients with high CD44v10 expression had higher histopathological grades and Tumor, Lymph Node, Metastasis stages, as well as shorter overall survival time, implying a potential role of CD44v10 in BrCas progression.

Accumulating evidences have indicated that CD44v10-containing isoforms, such as CD44v8-10, CD44v4-10, and CD44v3,8 to 10, are related to cancers in diversity (4, 5, 24). However, the function of CD44v10 alone was largely unknown. Given the fact that the knockdown of exon-v10 could downregulate all expressions of CD44v10-containing isoforms (7), we performed transcriptomic analysis by establishing a CD44v10 overexpression cell line instead of CD44v10 knockdown to search for mechanisms on CD44v10 in regulating BrCas progression. We found that the differentially expressed genes induced by ectopic expression of CD44v10 were mostly associated with glycolytic processes. We then confirmed that CD44v10 upregulation could promote glycolytic phenotypes, including glucose consumption and lactate production along with ROS defense, whereas CD44v10 downregulation reverses such effects. These findings were consistent with previous studies showing that CD44 is involved in the regulation of the glycolytic pathway in cancer cells (25, 26). To further investigate the deeper mechanism of CD44v10-mediated glycolysis, we focused on glycolysis-related genes. After transcriptomic analysis of TNBC cells, we identified that GLUT1 was the most robustly upregulated gene in CD44v10 overexpression cells. As illustrated before, GLUT1 is the main transporter for cellular glucose uptake and has been reported to be significantly elevated in TNBCs (27). Our study firstly connected GLUT1 with CD44v10 for the findings that inhibiting GLUT1 could attenuate CD44v10-enhanced glycolysis in TNBC cells. However, it should be noted that GLUT1 downregulation did not completely disrupt the glycolytic phenotype, we hypothesized that CD44v10 may facilitate glycolysis through upregulation of GLUT1 expression along with other genes which need further confirmation. In supporting with our results, a previous study indicated that CD44 ablation could suppress GLUT1 expression (25) in which the underlying mechanism has not been clarified. Our lab recently reported that CD44v10 could activate MAPK/ERK and PI3K/AKT signaling pathways (7). Meanwhile, others demonstrated that the induction of GLUT1 was associated with the above signaling (17, 28). Therefore, we questioned whether CD44v10 could upregulate GLUT1 by triggering MAPK/ERK and PI3K/AKT pathways. Consistent with our hypothesis, the inhibition of the pathways significantly reduced CD44v10-induced GLUT1 protein expression, implying that CD44v10 may upregulate GLUT1 through the activation of MAPK/ERK and PI3K/AKT signaling pathways.

It has been well accepted that metabolic reprogramming is one of the hallmarks of cancer (29). Recent studies suggested that CSCs may have higher glycolytic activity compared with the bulk of the general cancer cells (12, 13). As CD44v8-10 and CD44v4-10 could facilitate CSC activities in gastric cancer and intestinal cancer (5, 24), we wonder whether CD44v10 function a crucial role in the regulation of TNBC stemness properties independently. Our following experiments revealed that elevated CD44v10 was not only enriched in spheres of TNBC cells but was also essential to inducing a stem cell-like state. These findings suggested that CD44v10 alone may confer stemness properties and potentially act as a marker of CSCs in TNBC. Notably, we also demonstrated that the disruption of GLUT1 decreases glycolytic activity and thereby depresses the enhanced stemness induced by CD44v10 upregulation, providing a novel mechanism by which CD44 variants confer stem-like properties of cancer cells. In light of these observations, CD44v10 may individually promote stem cell-like properties of TNBC cells through GLUT1-mediated glycolysis.

A growing body of literature has indicated that CD44v-expressing cancer cells especially CSCs show inherent chemoresistance (30, 31). Given the potential role of CD44v10 in CSCs regulation, we next asked whether CD44v10 could play a role in mediating PTX sensitivity. We accordingly performed drug sensitivity experiments *in vitro* and found that TNBC cells treated with PTX expressed higher levels of CD44v10 along with CSC-related genes in a dose-dependent manner. Additionally, the upregulation of CD44v10 alone could inhibit the sensitivity to PTX-induced cell death, while downregulation of CD44v10 reverses this effect, suggesting the involvement of CD44v10 in the regulation of PTX sensitivity. In concordance with our results, a previous study indicated that CD44v8-10 attenuates apoptotic responses to cisplatin in urothelial cancer (6). Furthermore, other CD44 variant exons, such as CD44v6 and CD44v9, have also been described to confer chemotherapeutic resistance to cancer cells through the regulation of redox balance (32, 33, 34). In view of this, the results of our present study raise the possibility that CD44v10 potentiates the ability of TNBC cells to defend themselves against chemotherapy-induced ROS. Strikingly, we further confirmed that the attenuation of PTX sensitivity caused by CD44v10 overexpression could be rescued by GLUT1 downregulation. To this end, we proposed a notion that targeting CD44v10 may disrupt the glucose metabolism of CSCs and thereby improve the sensitivity of TNBC cells to therapeutics, which warrants further investigation.

In conclusion, our study unveiled the roles of CD44v10 alone in promoting stemness properties and regulating PTX sensitivity through GLUT1-mediated glycolysis, indicating that CD44v10 may be a therapeutic target to improve TNBC therapy.

## Experimental procedures

### Cell cultures and reagents

Human BrCa cell lines (MCF10A, MDA-MB-468, Hs578t, BT-549, and MDA-MB-231) were purchased from the Cell Bank of the Type Culture Collection of the Chinese Academy of Sciences. All human cell lines have been authenticated using STR profiling within the last 3 years. MCF10A cells were cultured in MEGM (Lonza, Thermo Fisher Scientific, Inc), BT-549 cells were cultured in RPMI-1640 medium (Gibco, Thermo Fisher Scientific, Inc), and MDA-MB-468, Hs578t, and MDA-MB-231 cells were cultured in high-glucose Dulbecco's modified Eagle's medium (Gibco, Thermo Fisher Scientific, Inc). All the media were supplemented with 10% FBS (Bovogen Biologicals Pty Ltd), 100 U/ml penicillin, and 100 mg/ml streptomycin. Additional insulin with a final concentration of 0.01 mg/ml was added to the culture medium of BT-549 and Hs-578t. All cell lines were maintained at a temperature of 37 °C in humidified air with 5% CO_2_. In addition, all cells were grown to 80% confluency for the experiments. All experiments were performed with mycoplasma-free cells. The ERK inhibitor U0126 (S1102) and PI3K inhibitor LY294002 (S1105) were purchased from Selleck. The standard stock and trial solutions were prepared according to the manufacturer’s instructions.

### Immunohistochemical analysis

Three independent tissue microarrays of human BrCas were purchased from Shanghai Outdo Biotech. To investigate the expression of CD44v10 in human BrCas, a tissue microarray (HBre-Duc060CS-03) including 25 paired available breast cancerous tissues and peritumoral tissues was examined by immunohistochemistry. To further evaluate the relevance of CD44v10 in different subtypes of BrCas, another cohort (HBreD080CS01) that consisted of 71 available breast primary tumor tissues (30 cases of luminal BrCas, 13 cases of BrCas with HER2 overexpression, and 28 cases of TNBCs) and six adjacent normal tissues was analyzed. Additionally, a tissue microarray (HBreD136Su02) containing 135 available BrCa patients was prepared to identify the correlation between CD44v10 and survival time. Immunohistochemical staining for CD44v10 was performed as reported previously (35). Briefly, these tissue sections were treated as follows: dewaxing, dehydration, antigen retrieval, and inhibition of endogenous peroxidase activity as well as nonspecific binding. Then, a mouse anti-human CD44v10 (1:200, Bio-Rad, MCA1733) antibody was added, and the slides were incubated at 4 °C overnight. After washing with PBS, the slides were incubated with a biotinylated secondary mouse antibody for 1 h, followed by streptavidin-ABC at room temperature. Then, the slides were developed with a 2,4-diaminobutyric acid Substrate Kit and counterstained with hematoxylin. The intensities of CD44v10 were quantitatively analyzed using IMAGE-PRO PLUS 6.0 software (Media Cybernetics). Mean density = IOD/area.

### Quantitative real-time PCR

Total RNA was extracted from cultured cells using TRIzol reagent. After quantification using a NanoDrop 2000 spectrophotometer, purified total RNA (1 μg) was reverse transcribed with the PrimeScript RT Reagent Kit with gDNA Eraser. Real-time PCR assays were performed by using SYBR Green Mix according to the manufacturer’s instructions. All quantitative real-time PCR values of each gene were normalized against that of β-actin. The relative expression of genes was calculated by the 2^-ΔΔCt^ method. The primer sequences for quantitative real-time PCR are listed in Table S1.

### Western blotting

RIPA buffer (Beyotime) was used for protein extraction. After the total protein concentration was determined by a bicinchoninic acid protein assay kit (Thermo Fisher Scientific, Inc), 20 μg protein samples were separated by 8% SDS polyacrylamide gels and transferred onto PVDF membranes (Millipore). The membranes were blocked with 5% skimmed milk in 0.1% TBS-Tween-20 at room temperature for 1 h and incubated with following primary antibodies overnight at 4 °C: CD44v10 (1:1000, Sigma-Aldrich, AB2082), pan-CD44 (1:1000, Abcam, ab189524), GLUT1 [1:1000, Cell Signaling Technology, (CST), 73,015], Oct4 (1:1000, CST, 2750), c-Myc (1:1000, CST, 5605), Klf4 (1:1000, CST, 4038), ALDH1 (1:1000, CST, 12,035), Nanog (1:1000, CST, 4903), p-ERK (1:2000, CST, 4370), ERK (1:1000, CST, 4695), p-AKT (1:1000, CST, 4060), AKT (1:1000, CST, 4691), and β-actin (1:1000, CST, 3700). On the following day, horseradish peroxidase-conjugated secondary antibodies (1:5000, Lianke Biotech Co., Ltd) were added. Then, the protein bands were subsequently captured using the enhanced plus chemiluminescence assay (Pierce) and were measured on an ImageQuant LAS 4000 mini.

### Plasmids and lentivirus

The siRNA constructs targeting human CD44v10 (NM_001202555) expression were designed and synthesized by RiboBio company (Guangzhou, China), and transfection was performed with riboFECT according to the manufacturer’s protocol. Sequences for siRNA knockdown, including CD44v10 siRNA (5′-CUACUUUACUGGAAGG UUA-3′), GLUT1 siRNA (5′-CUGUGGGCCUUUUCGUUAA-3′), and the scramble negative control siRNA sequence was 5′-UUCUCCGAACGUGUCACGU-3’. CD44v10 siRNA construct was inserted into the lentiviral vector hU6-MCS-CBh-gcGFP-IRES-puromycin (Genechem Company) for lentivirus production. Human CD44v10 was constructed by PCR-based amplification and cloned into the Ubi-MCS-3FLAG-SV40-Cherry-IRES-puromycin vector system (Genechem Company). Cells were infected with concentrated lentivirus according to the manufacturer’s instructions and treated with puromycin (2 μg/ml; Santa Cruz Biotechnology) for 3 weeks to generate stable CD44v10-knockdown or CD44v10-overexpression cells. The knockdown efficiencies and stable overexpression were verified by Western blotting assay.

### RNA sequencing and data analysis

Total RNA was extracted using TRIzol reagent (Invitrogen) according to the manufacturer’s protocol. The RNA libraries were then sequenced using an Illumina Novaseq 6000 instrument at the OE Biotech Co., Ltd. FPKM (Fragments Per kb Per Million Reads) of each gene was calculated using Cufflinks, and the read counts of each gene were obtained by HTSeq-count. Differential expression analysis was performed using the DESeq (2012) R package. We utilized a fold change > 1.5 with *p*-value < 0.05 as the threshold for significantly differential expression. Gene ontology enrichment and KEGG pathway enrichment analysis of differentially expressed genes were performed respectively using R based on the hypergeometric distribution. Gene set enrichment analysis (https://www.broadinstitute.org/gsea/index.jsp) was performed to analyze whether a set of genes show statistically significant differences between CD44v10 overexpression and vehicle control groups.

### Measurements of glucose consumption, lactate production, and ROS

Glucose and lactate concentrations of the cultured medium were measured by using a Glucose Assay kit-WST and Lactate Assay kit-WST (Dojindo Laboratories) according to the manufacturer’s instruction. Intracellular levels of ROS were determined by using 2′,7′-dichlorofluorescein-diacetate (Beyotime Biotech) according to the manufacturer’s instructions.

### Sphere formation assay

Cells were seeded into ultralow attachment 6-well plates (Corning) at a density of 1 × 10^4^ cells per well and then cultured in Dulbecco's modified Eagle's medium/F12 medium supplemented with B27 (1:50, Invitrogen), 20 ng/ml human recombinant epidermal growth factor (Peprotech), and 20 ng/ml human recombinant basic fibroblast growth factor (Peprotech) for 14 days. Cells were maintained in 5% CO_2_ at 37 °C, and the medium was replaced every 3 days. The total number of spheres greater than 50 μm in diameter was counted under an inverted microscope (Olympus Corporation).

### Aldefluor assay

The ALDEFLUOR kit (StemCell Technologies) was used to measure intracellular ALDH enzymatic activity. Briefly, one million cells were suspended in ALDEFLUOR assay buffer containing the ALDH substrate and incubated at 37 °C for 45 min. As a negative control, an aliquot of each cell sample was treated with diethylaminobenzaldehyde, a specific ALDH inhibitor. Stained cells were analyzed on the FACS CytoFLEX flow cytometer (Beckman-Coulter, Inc), and data analysis was performed using Cytexpert software 2.4 (Beckman-Coulter, Inc).

### CCK-8 toxic assay and colony formation experiment

The sensitivity of cells to PTX was measured by CCK-8 assay (Dojindo Laboratories). Cells were seeded in 96-well plates at a density of 3000 cells per well and then treated with various concentrations (2.5 nM, 5 nM, 10 nM 20 nM, or 40 nM) of PTX (MedChemExpress) after the cells were attached. Then incubated for 48 h, replaced fresh culture medium, and added CCK-8 reagent to each well according to the manufacturer’s protocol, incubated at 37 °C for an additional 2 h. Absorbance was measured at 450 nm using a microplate absorbance reader (Bio-Rad Laboratories, Inc). Colony formation assay of BrCas cells was carried out by plating infected cells at a density of 1000 cells/well in a 6-well plate, and 2 nM of PTX was added. After 2 weeks of incubation, cells were washed three times with PBS, fixed with 4% paraformaldehyde for 30 min at room temperature, followed by staining with 0.1% crystal violet (BaSO Biotech Co., Ltd) for 30 min at room temperature. After rinsing three times, the stained colonies were imaged, and the number of colonies was counted by the naked eye.

### Statistical analysis

Statistical analysis was performed using GraphPad Prism 8.0.2 (GraphPad Software, Inc). Data are presented as the group mean ± standard deviation (SD). Statistical analysis was performed using an unpaired Student’s *t* test for two-group comparison and a one-way analysis of variance (ANOVA) for multigroup comparisons. Differences in survival were calculated by the log-rank Mantel-Cox test. The *p*-value <0.05 was considered to be statistically significant (∗*p* <0.05, ∗∗*p* <0.01, ∗∗∗*p* <0.001). Generally, all experiments were carried out with triplicate independent replicates.

## Data availability

The data that support the findings of this study are available in the methods and/or supplementary material of this article.

## Supporting information

This article contains supporting information.

## Conflicts of interest

The authors declare no conflict of interest with the contents of the article.
