# Supplementary material for: Cell adhesion molecule CD44v10 promotes stem-like properties in triple-negative breast cancer cells via glucose transporter GLUT1-mediated glycolysis
Source: J Biol Chem. 2022 Oct 12;298(11):102588. doi: 10.1016/j.jbc.2022.102588 (PMC9647553; doi:10.1016/j.jbc.2022.102588)
Supplement: Supplemental data [file mmc1.docx]

**Cell adhesion molecule CD44v10 promotes stem-like properties in triple-negative breast cancer cells via glucose transporter GLUT1-mediated glycolysis**

Qian Guo^1,2†^, Yaqi Qiu^1,2†^, Yiwen Liu^1^, Yiqing He^1^, Guoliang Zhang^1^, Yan Du^1^, Cuixia Yang^1,2^, and Feng Gao^1,2*^


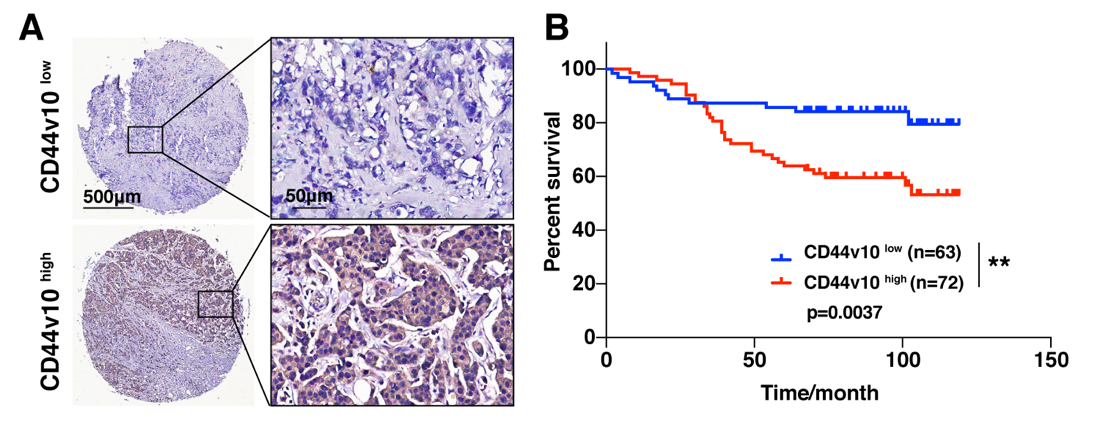


**Figure S1.** **Kaplan-Meier analysis of the** **overall survival of BrCa patients with different CD44v10 expression levels**

(A). Representative images of BrCa tissues with low and high CD44v10 expression are shown by IHC (Brown: CD44v10). (B). Kaplan–Meier curves showed the survival of 135 BrCa patients with different CD44v10 expression levels: CD44v10 ^low^ (blue line, n=63) and CD44v10 ^high^ (red line, n=72). ***p* <0.01.


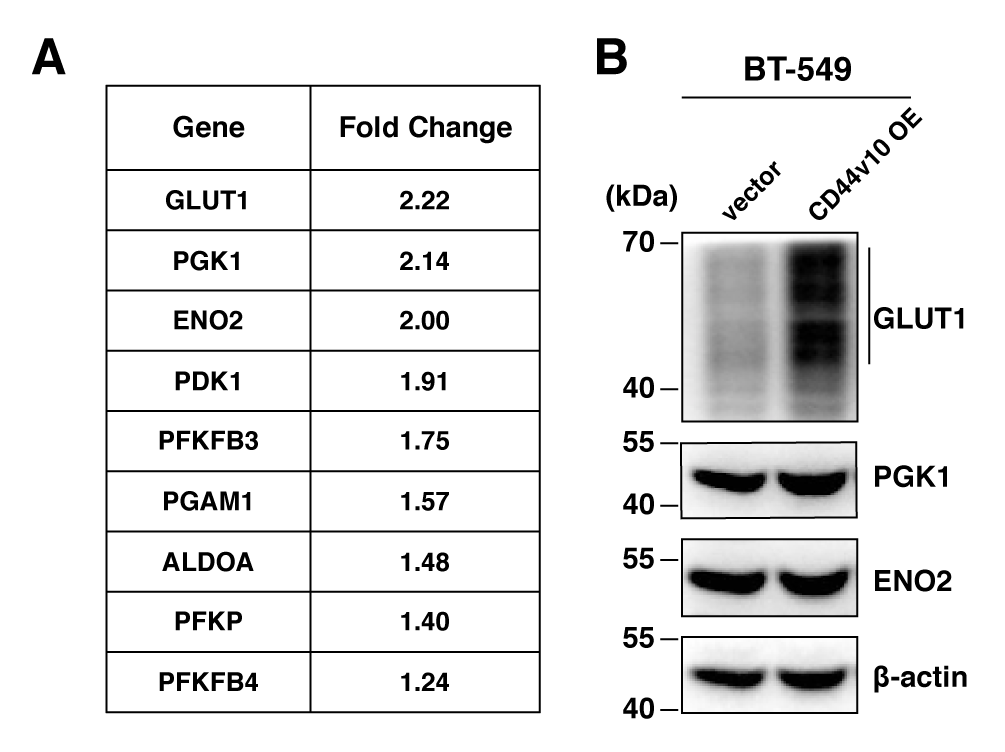


**Figure S2.** **The expressions of glycolysis-related genes in CD44v10 overexpression cells compared with control group**

(A) The transcriptional levels of glycolysis-related genes in BT549 CD44v10 overexpression and control cells by qPCR assay. (B) The protein levels of the robustly up-regulated candidates (GLUT1, PGK1, ENO2) upon CD44v10 ectopic expression.


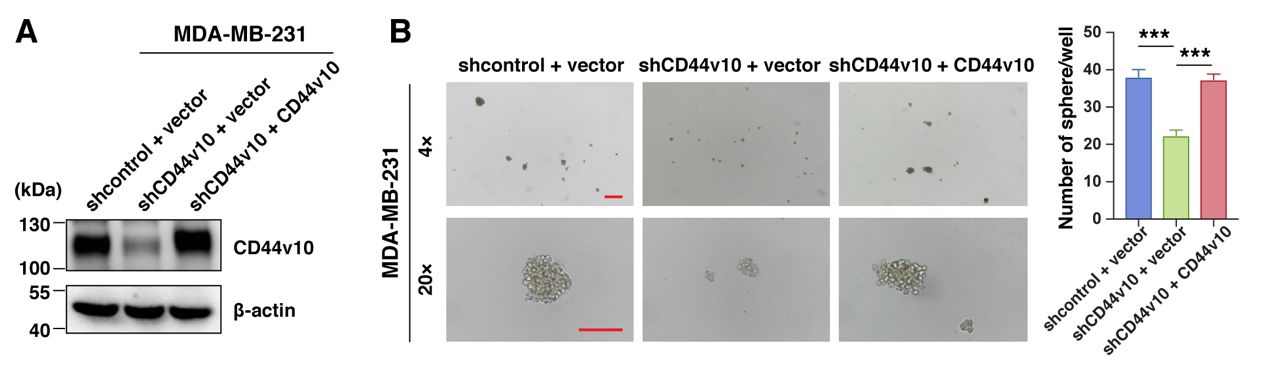


**Figure S3.** **The effects of rescued CD44v10 expression on sphere-forming ability of TNBC cells**

(A) Knockdown and re-expression efficiency of CD44v10 in MDA-MB-231 cells were evaluated by western blotting. (B) The effects of CD44v10 knockdown and re-expression on sphere-forming ability. Scar bars, 400 μm for (4×) and 200 μm for (20×). Data are shown as the mean ± SD. ****p* <0.001.


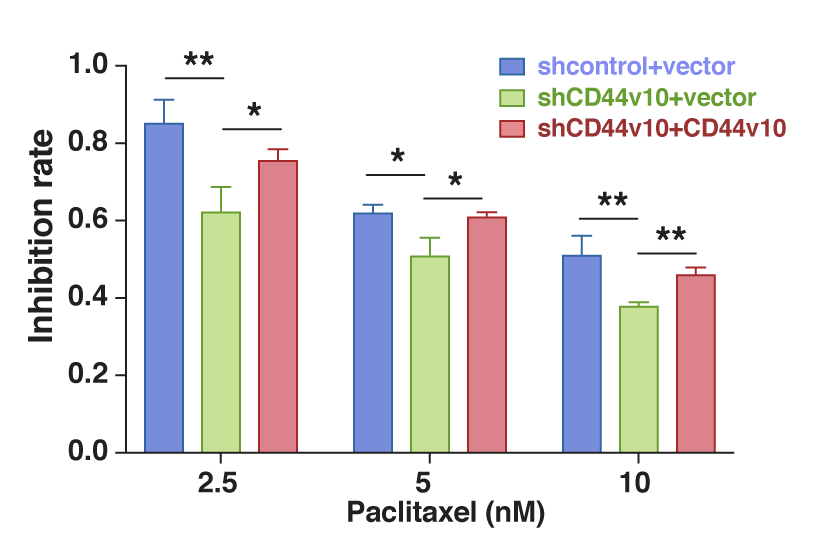


**Figure S4.** **The effects of rescued CD44v10 expression on paclitaxel sensitivity of TNBC cells**

CCK-8 assay was used to evaluate the chemo-sensitivity of MDA-MB-231 cells to different concentrations of paclitaxel after 48 h treatment. Data are shown as the mean ± SD. **p* <0.05, ***p* <0.01.

| Table S1. List of Primer sequences used for PCR | |
| --- | --- |
| Gene | Sequences |
| CD44v10-qPCR-F | ACAGAATCCCTGCTACCAATAGG |
| CD44v10-qPCR-R | TGGAATGTGTCTTGGTCTCCTG |
| GLUT1-qPCR-F | GAACTCTTCAGCCAGGGTCC |
| GLUT1-qPCR-R | ACCACACAGTTGCTCCACAT |
| ALDOA-qPCR-F | GACACTCTACCAGAAGGCGGAT |
| ALDOA-qPCR-R | GGTGGTAGTCTCGCCATTTGTC |
| ALDOC-qPCR-F | TCACGTAGCTCTGCGACATC |
| ALDOC-qPCR-R | CAGAAAGGGCTGGGTACGAG |
| PDK1-qPCR-F | GACCGAGGAGGTGGCGTTCC |
| PDK1-qPCR-R | AAAACCAGCCAGAGGCACTGCG |
| PFKFB3-qPCR-F | GGCAGGAGAATGTGCTGGTCAT |
| PFKFB3-qPCR-R | CATAAGCGACAGGCGTCAGTTTC |
| PFKFB4-qPCR-F | GGGTGCCTCTTGGCCTTAAA |
| PFKFB4-qPCR-R | GCCCACACGGCATACTTTTC |
| PFKP-qPCR-F | AGGCAGTCATCGCCTTGCTAGA |
| PFKP-qPCR-R | ATCGCCTTCTGCACATCCTGAG |
| PGAM1-qPCR-F | GCTCTGCCCTTCTGGAATGAAG |
| PGAM1-qPCR-R | ATACCAGTCGGCAGGTTCAGCT |
| PGK1-qPCR-F | CCGCTTTCATGTGGAGGAAGAAG |
| PGK1-qPCR-R | CTCTGTGAGCAGTGCCAAAAGC |
| ENO1-qPCR-F | AGTCAACCAGATTGGCTCCGTG |
| ENO1-qPCR-R | CACAACCAGGTCAGCGATGAAG |
| ENO2-qPCR-F | CTGTATCGCCACATTGCTCAGC |
| ENO2-qPCR-R | AGCTTGTTGCCAGCATGAGAGC |
| ACTB-qPCR-F | CACCATTGGCAATGAGCGGTTC |
| ACTB-qPCR-R | AGGTCTTTGCGGATGTCCACGT |
